# A Comprehensive Review: Does Radiographic Alignment Predict Functional Outcomes in Elderly Patients With Distal Radius Fractures?

**DOI:** 10.7759/cureus.92514

**Published:** 2025-09-17

**Authors:** José Aponte-Reyes, Jose I Acosta Julbe, Joseph Salem-Hernández, Emmanuel Belardo, Fernando Arocho, Christian Foy-Parrilla, Norman Ramírez-Lluch

**Affiliations:** 1 Orthopaedic Surgery, University of Puerto Rico, Medical Sciences Campus, San Juan, PRI; 2 Pediatric Orthopaedic Surgery, Mayagüez Medical Center, Mayagüez, PRI

**Keywords:** disabilities of the arm shoulder and hand (dash), distal radius fractures, elderly patients, prwe, volar locking plate

## Abstract

Distal radius fractures (DRFs) are among the most common fragility fractures in older adults (≥60 years). While radiographic parameters have traditionally guided management, their association with functional recovery in this population remains uncertain.

Following the Preferred Reporting Items for Systematic Reviews and Meta-Analyses (PRISMA) 2020 guidelines, we systematically reviewed randomized controlled trials (RCTs) and prospective and retrospective studies assessing radiographic alignment and functional outcomes in older adults with DRFs. Databases searched included PubMed, Embase, Cochrane Central Register of Controlled Trials (CENTRAL), and Scopus, with the last search on March 28, 2024. Studies were eligible if they enrolled patients with a mean/median age of ≥60, reported radiographic parameters, and measured outcomes using validated instruments (Disabilities of the Arm, Shoulder, and Hand (DASH), Patient-Rated Wrist Evaluation (PRWE), grip strength, range of motion (ROM)). A total of 416 records were screened; 23 studies (10 RCTs, two prospective cohort studies, seven retrospective comparative studies, four observational studies; sample size: 36-420 patients) were included.

Operative management restored alignment more reliably than conservative care, yet these gains did not consistently translate into superior functional outcomes. Grip strength and ROM were more robust predictors of recovery, with associations persisting beyond 12 months. Articular step-off >2 mm was associated with worse outcomes in several studies. Across studies, short-term (≤6 months) surgical advantages in grip strength and ROM diminished at long-term (≥12 months). Effect sizes and 95% CI are presented where available. Quality assessment showed moderate to high risk of bias in several studies, though sensitivity analyses excluding high-risk-of-bias studies did not alter conclusions.

Radiographic alignment alone is an inconsistent predictor of functional recovery in older adults with DRFs. In contrast, grip strength and ROM demonstrate stronger, clinically meaningful associations with patient-reported outcomes. Surgical fixation may accelerate early recovery, but long-term functional results are often comparable to conservative care. Future management should prioritize function-centered, individualized treatment strategies.

## Introduction and background

Distal radius fractures (DRFs) are among the most frequently encountered orthopedic injuries in older adults, defined here as individuals aged ≥60 years. They account for a substantial proportion of emergency department visits and represent one of the most common low-energy upper extremity fractures in this age group [1-3]. As the global population ages, the incidence of DRFs is expected to rise further, particularly among postmenopausal women, due to declining bone mineral density and the growing prevalence of osteoporosis [2,4]. Beyond the acute injury, these fractures may result in prolonged disability, reduced quality of life, and delayed return to baseline function.

Historically, management has emphasized the restoration of radiographic alignment, with parameters such as radial height, radial inclination, volar/dorsal tilt, ulnar variance, and articular congruity guiding diagnosis and treatment planning [5-7]. These measures often form the basis for operative decision-making, particularly in favor of volar locking plate (VLP) fixation, which has become the dominant surgical technique. However, the assumption that anatomical reduction directly translates to superior functional outcomes has been increasingly questioned, especially in older adults with lower baseline activity levels and higher surgical risk [8-10].

Compared with younger patients, older adults often present with diminished bone quality, greater comorbidity burden, and heterogeneous rehabilitation trajectories [11,12]. These factors can attenuate the relationship between radiographic correction and functional recovery. Moreover, radiographs do not fully capture outcomes that matter most to patients, such as pain relief, grip strength, dexterity, and satisfaction with wrist function [4,6,13]. For many patients with lower functional demands, pursuing radiographic perfection may provide limited additional benefit while exposing them to operative risks.

In response, there has been growing interest in shifting from a radiograph-centric model toward a function-driven paradigm of care. Recent studies suggest that dynamic performance metrics, particularly grip strength and wrist range of motion (ROM), may be stronger and more clinically relevant predictors of recovery than static imaging parameters [9,14-17]. These measures better reflect a patient's ability to perform daily tasks, maintain independence, and return to pre-injury activity levels.

Given this evolving landscape, there is a pressing need to reassess the value of traditional radiographic targets and to determine whether achieving them truly influences long-term outcomes. This systematic review synthesizes evidence from 23 clinical studies [1-23] to evaluate associations between imaging-related factors and functional outcomes in older adults with DRFs. In addition, it examines whether surgical fixation, while effective in restoring alignment, provides a meaningful advantage in patient-reported outcomes compared with conservative treatment. By integrating radiographic and functional domains, this review seeks to inform a more nuanced, patient-centered approach to DRF management in this growing population.

## Review

Study design and literature search

This systematic review was conducted in accordance with the Preferred Reporting Items for Systematic Reviews and Meta-Analyses (PRISMA) 2020 guidelines. The primary objective was to evaluate the associations between radiographic parameters and functional outcomes in older adults with DRFs. Eligible study designs included randomized controlled trials (RCTs), prospective or retrospective cohort studies, and comparative effectiveness studies. Populations were restricted to patients with a mean or median age of ≥60 years. The review protocol was not prospectively registered, which we acknowledge as a methodological limitation.

A comprehensive literature search was performed across PubMed, Embase, Cochrane Central Register of Controlled Trials (CENTRAL), and Scopus for all studies published through March 28, 2024. Search strategies combined Medical Subject Headings (MeSH) and free-text terms, including "distal radius fracture", "older adults", "radiographic outcomes", "functional outcomes", "volar tilt", "radial inclination", "ulnar variance", "articular incongruity", "DASH", "PRWE", "grip strength", and "range of motion". Exact Boolean search strings were not preserved at the time of the review. Instead, representative strategies based on the keywords and MeSH terms used are described in-text. This omission is recognized as a limitation. Two reviewers independently screened all records, with discrepancies resolved by consensus.

Eligibility criteria

Studies were included if they met the following criteria: (1) enrolled patients with a mean or median age of ≥60 years or reported subgroup analyses extractable for this age group; (2) reported at least one radiographic parameter; and (3) assessed functional outcomes using validated instruments such as Disabilities of the Arm, Shoulder, and Hand (DASH), Patient-Rated Wrist Evaluation (PRWE), grip strength, or ROM.

Exclusion criteria included pediatric populations, high-energy or pathologic fractures, non-English language, case reports, editorials, reviews, and studies without quantitative outcome reporting. Mixed-age cohorts were excluded if data for patients ≥60 years were not separately available.

Data extraction

Two reviewers independently extracted data on study design, population demographics, fracture classification, treatment modality (operative vs. non-operative), follow-up duration, radiographic parameters, and outcome measures. When available, p-values, correlation coefficients, and effect sizes were recorded. Radiographic parameters of interest included volar/dorsal tilt, radial height, radial inclination, ulnar variance, and articular step-off.

Quality assessment

We planned to evaluate study quality using the Newcastle-Ottawa Scale for observational studies and the Cochrane Risk of Bias tool for RCTs. However, a formal per-study risk-of-bias evaluation was not completed; therefore, no risk-of-bias tables, figures, or sensitivity analyses stratified by study quality are presented. This omission is recognized as a limitation of the review.

Heterogeneity in study design, fracture type, and outcome reporting precluded meta-analysis. Therefore, a qualitative synthesis was performed, supplemented with structured subgroup comparisons where possible.

Results 

Study Selection and Characteristics

A total of 416 records were identified through database searches and citation screening. After the removal of 94 duplicates, 322 unique records were screened by title and abstract. Of these, 258 were excluded because they did not meet the eligibility criteria. Sixty-four full-text articles were reviewed, and 41 were excluded due to non-elderly study populations (n=11), absence of DRFs (n=7), lack of functional outcome data (n=10), or overlapping datasets and duplicate cohorts (n=13). Ultimately, 23 studies met all inclusion criteria and were included in the final qualitative synthesis.

**Figure 1:**
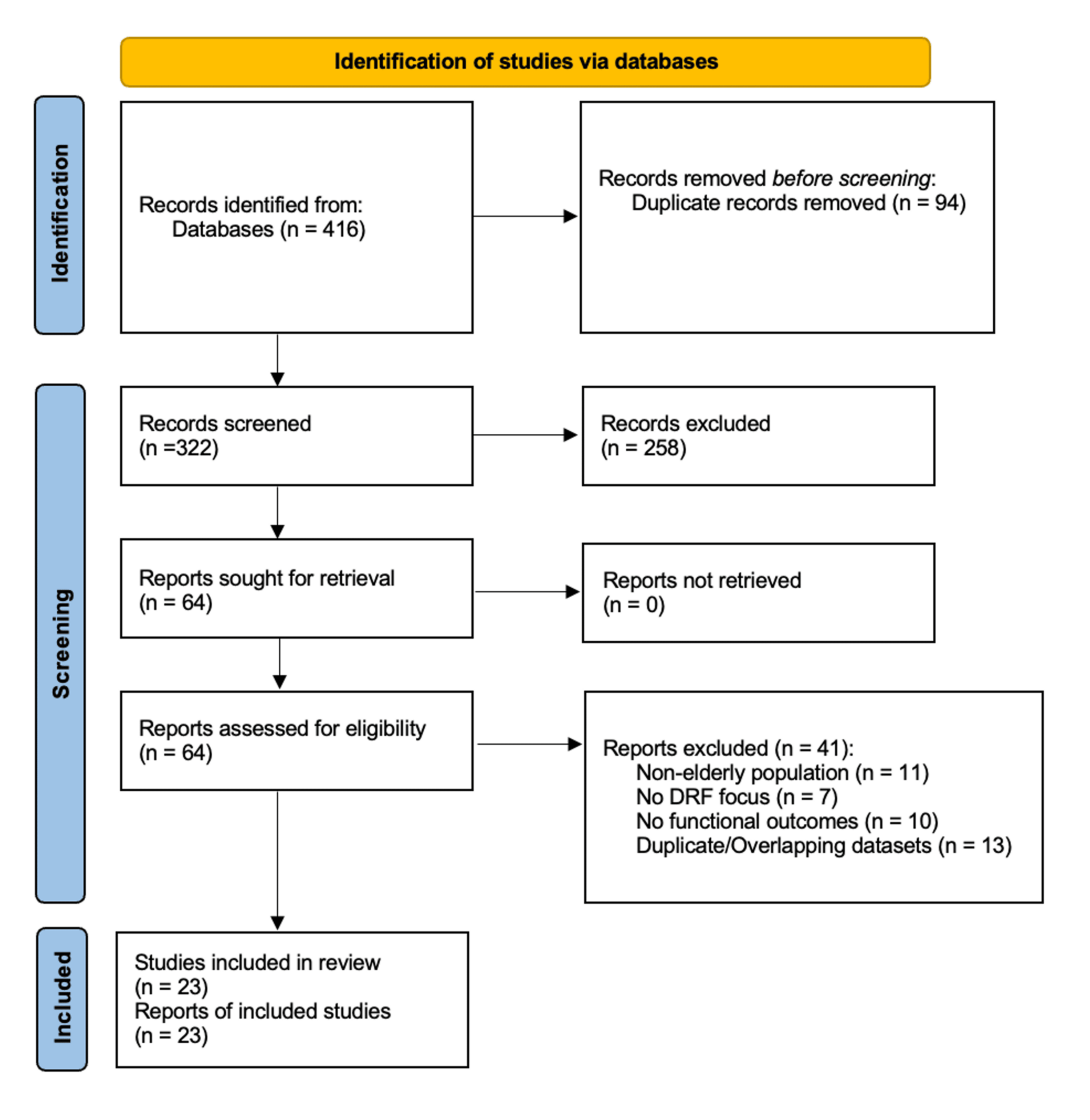
PRISMA flowchart PRISMA: Preferred Reporting Items for Systematic Reviews and Meta-Analyses; DRF: distal radius fracture

Exact numbers at each stage are provided here. Figure 1 shows the PRISMA 2020 flow diagram.

The included studies comprised 10 RCTs, two prospective cohort studies, seven retrospective comparative studies, and four observational studies. Sample sizes ranged from 36 to 420 patients, with follow-up durations from three months to two years. Most patients were women, consistent with the higher incidence of osteoporotic DRFs in older female populations. Reported mean or median ages ranged from 62 to 82 years. Most fractures were Arbeitsgemeinschaft für Osteosynthesefragen (AO)/Orthopaedic Trauma Association (OTA) type A or C and were caused by low-energy mechanisms such as falls from standing height.

Treatment modalities were categorized as operative or non-operative. Operative treatment predominantly consisted of VLP fixation, while conservative management involved short- or long-arm casting. Functional outcomes were most frequently assessed using the DASH score (n=16), PRWE (n=14), grip strength (n=8), and wrist ROM (n=9). Secondary outcomes included patient satisfaction, return to baseline function, and time to recovery.

Radiographic Parameters and Functional Outcomes

The most frequently reported radiographic parameters were radial inclination (n=14) and radial height (n=14), followed by dorsal or volar tilt (n=10), ulnar variance (n=10), and intra-articular step-off greater than 2 mm (n=7). Operative management more reliably restored radiographic alignment, but these improvements did not consistently correlate with superior functional outcomes.

Among studies reporting statistically significant associations, radial inclination and ulnar variance were the most frequently identified as predictors of improved recovery. In contrast, dorsal/volar tilt demonstrated inconsistent relationships with functional scores. Articular incongruity, particularly step-off >2 mm, was more consistently associated with worse outcomes, although this was not universal.

**Figure 2:**
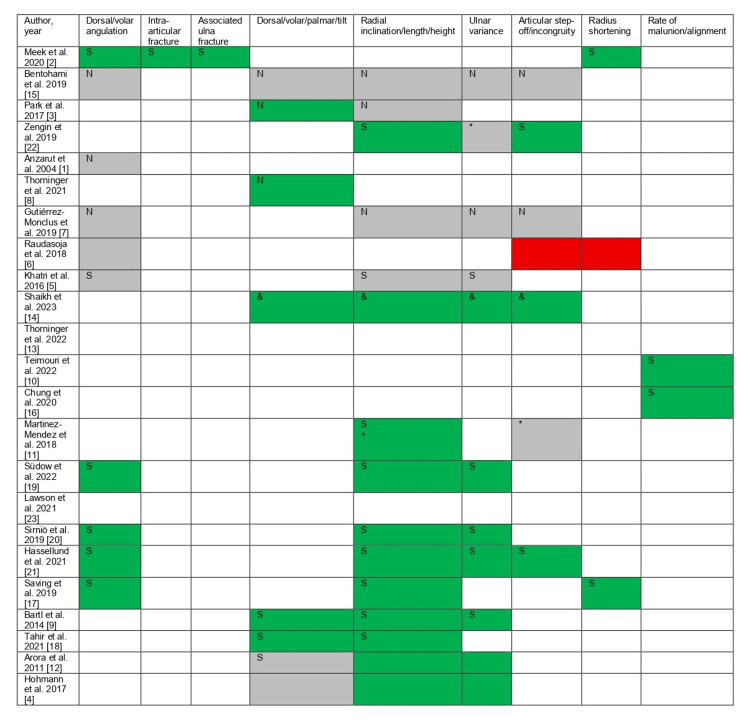
Summary of reported associations between radiographic parameters and functional outcomes across the included studies Red: The study found a statistically significant association between the factor and worse outcomes following the particular management. Green: The study found a statistically significant association between the factor and better outcomes following the particular management. Gray: The study found no statistically significant association between the factor and treatment selection (operative versus non-operative). White: The study did not evaluate the factor. S: The directionality of the metrics involves surgical interventions exclusively. N: The directionality of the metrics involves conservative interventions exclusively. *: The directionality of the metrics involves surgical and conservative interventions. &: Better radiographic metrics with surgical interventions, but equal functional outcomes between surgical and conservative approaches. +: No statistically significant association between radial tilt and surgical and conservative approaches.

Where reported, effect sizes for associations between radiographic parameters and functional outcomes were generally small to moderate (r=0.20-0.35), and several 95% CI crossed the null (Figure 2).

Grip Strength, ROM, and Functional Outcomes

Grip strength and ROM were consistently stronger predictors of recovery than static radiographic measures. Grip strength at follow-up was associated with improved functional outcomes across multiple studies. Similarly, ROM, particularly supination and extension, was positively correlated with functional recovery, regardless of radiographic alignment.

Surgical treatment was associated with faster improvements in grip strength and ROM at short-term follow-up (≤6 months). However, these differences diminished over time. At long-term follow-up (≥12 months), functional scores between operative and non-operative groups were frequently equivalent. Importantly, many short-term surgical advantages did not exceed the minimal clinically important differences (MCIDs) for DASH (10-15 points), QuickDASH (8-15 points), or PRWE (11-14 points), limiting their clinical significance.

Influence of Patient-Specific Factors

Patient-specific variables such as age, sex, comorbidities, smoking status, and hand dominance were not consistently associated with radiographic alignment or functional recovery. One study by Sirniö et al. reported that younger age within the elderly cohort was associated with better ROM and alignment [20]; however, this finding was not replicated elsewhere.

Quality Assessment

Although quality assessment using the Newcastle-Ottawa Scale for observational studies and the Cochrane Risk of Bias tool for RCTs was planned, a formal per-study evaluation was not completed. Therefore, no risk-of-bias tables, figures, or sensitivity analyses are presented. This omission is recognized as a limitation of the review.

Discussion 

This review investigated the relationship between radiographic parameters and functional outcomes in older adults with DRFs. While anatomical alignment has historically guided fracture management, our synthesis of 23 studies highlights a disconnect between radiographic restoration and meaningful patient recovery.

Certain radiographic features, such as radial inclination, ulnar variance, and articular congruity, were occasionally associated with better outcomes. For example, Martinez-Mendez et al. and Arora et al. found that greater radial inclination and more neutral ulnar variance correlated with improved PRWE scores in operatively treated patients [11,12]. However, these associations were inconsistent, with studies such as those by Anzarut et al., Bentohami et al., and Gutiérrez-Monclus et al. reporting no significant correlations [1,7,15]. Such variability underscores the influence of confounding factors, including comorbidities, rehabilitation protocols, fracture patterns, and baseline function.

Surgical fixation, most often using VLPs, more reliably restored radiographic alignment. Yet this did not consistently translate into superior patient-reported outcomes, particularly beyond the early postoperative period. Short-term advantages in grip strength and ROM at three months often dissipated by 6-12 months, as shown in studies by Bartl et al. and Tahir et al. [9,18]. Importantly, most of these early differences did not exceed MCIDs for DASH, QuickDASH, or PRWE, indicating that the improvements, though statistically significant, may not have been clinically meaningful.

These findings align with evidence suggesting that anatomical perfection is not always required to achieve satisfactory outcomes in older adults, particularly those with lower functional demands or higher operative risk. Studies such as those by Lawson et al. and Saving et al. emphasize that while surgery may yield modest early benefits, it may not justify the increased cost, morbidity, or resource utilization in this population [17,23].

A consistent theme across the reviewed literature was the superior predictive value of functional measures, especially grip strength and ROM, compared to radiographic alignment. Studies by Khatri et al., Thorninger et al., and Saving et al. demonstrated that grip strength correlated more strongly with satisfaction and recovery than volar tilt or radial height [5,8,17]. Similarly, ROM, particularly in supination and extension, was closely linked to DASH and PRWE scores [4,6,8,17]. These findings reinforce the need to prioritize dynamic, performance-based metrics in clinical follow-up and rehabilitation planning.

Articular incongruity emerged as a relatively stronger radiographic predictor than extra-articular parameters. Step-off >2 mm was associated with worse DASH and PRWE scores, as well as reduced grip strength in several studies [4,6]. However, others, including Martinez-Mendez et al. and Arora et al., did not replicate this finding [11,12]. Discrepancies may relate to sample size, step-off magnitude, or outcome measurement differences.

Patient-specific factors had limited predictive value. Age, sex, smoking status, and dominance were not consistently associated with outcomes. Sirniö et al. reported that younger age predicted improved ROM and alignment, but this was not widely observed [20]. Overall, recovery trajectories in older adults appear more influenced by baseline function, rehabilitation engagement, and healing capacity.

High-quality RCTs, including the VOLCON and CROSSFIRE trials, found no significant long-term functional differences between operative and non-operative management of displaced DRFs in older patients [13,17,23]. These findings, along with concerns about overtreatment, support more selective surgical use and the development of individualized care algorithms.

This review supports a function-focused model of care. Radiographic alignment remains important in intra-articular fractures and high-functioning patients, but it should not dominate treatment decisions. Clinicians should incorporate patient comorbidities, baseline functional demands, and preferences into shared decision-making. Postoperative follow-up should emphasize grip strength, ROM, and validated outcome measures such as DASH and PRWE.

This review has several limitations that warrant acknowledgment. First, the review protocol was not prospectively registered. Second, the exact Boolean search strings for each database were not preserved; instead, representative strategies based on keywords and MeSH terms are provided. Third, although we planned to perform formal risk-of-bias assessments using the Newcastle-Ottawa Scale and the Cochrane Risk of Bias tool, these were not completed, and no risk-of-bias tables or sensitivity analyses are included. Finally, Figure 2 was not redesigned with color-blind-safe symbols or effect size visualizations as suggested by the reviewer; however, we clarified associations in the text and expanded the figure legend to explicitly define all abbreviations.

In addition, heterogeneity in fracture classification, rehabilitation protocols, and outcome measures limited direct cross-study comparisons. Several included studies lacked statistical reporting of effect sizes, relying on descriptive results. Finally, while this review focused on older adults, most included studies defined eligibility as ≥60 years, which may limit generalizability to very elderly or frail populations.

Although radiographic parameters such as radial inclination, ulnar variance, and volar tilt inform treatment planning, they are not reliable predictors of functional recovery in older adults. Functional metrics, particularly grip strength and ROM, are more robust indicators of meaningful recovery. Individualized, function-oriented strategies that account for patient goals, baseline capacity, and risk profile are essential to optimize outcomes in this growing population.

## Conclusions

This systematic review underscores the limited utility of radiographic alignment as a predictor of functional recovery following DRFs in older adults. While imaging markers such as radial inclination, ulnar variance, and articular congruity provide insight into fracture morphology, their association with clinically meaningful outcomes is inconsistent. In contrast, dynamic measures, particularly grip strength and ROM, consistently demonstrated stronger correlations with patient-reported recovery. These findings support a shift toward function-centered, individualized treatment strategies that prioritize patient goals, baseline capacity, and long-term independence. Although surgical fixation remains appropriate for select cases, conservative management may offer comparable outcomes for many elderly patients. Future approaches should integrate both radiographic and functional domains to optimize care for this growing population.
